# New correlations between ocular parameters and disease severity in Spanish patients with Gaucher’s disease Type I

**DOI:** 10.1371/journal.pone.0260241

**Published:** 2021-12-06

**Authors:** Olivia Esteban, Miguel Angel Torralba, Susana Olivera, Mireya Martinez, Paula Montes, Sara Marco, Javier Ascaso

**Affiliations:** 1 Department of Ophthalmology, “Lozano Blesa” University Hospital, Zaragoza, Spain; 2 Aragon Health Research Institute (IIS Aragon), Zaragoza, Spain; 3 Department of Internal Medicine, “Lozano Blesa” University Hospital, Zaragoza, Spain; 4 Department of Ophthalmology, San Pedro Hospital, Logroño, Spain; UPMC and Pittsburgh School of Medicine, UNITED STATES

## Abstract

**Background:**

Gaucher’s disease is associated with a high variety of structural and functional abnormalities in the eye, which do not always affect visual acuity. The purpose of this study was to analyse ocular features in Spanish patients with Gaucher’s disease type I, and to investigate their possible correlation with phenotypic and burden parameters of this entity.

**Methods:**

This cross-sectional observational study compared parameters belonging to 18 eyes from 9 Spanish patients with Gaucher’s disease Type I with 80 eyes from 40 healthy controls. Complete ophthalmological examination included choroidal and retinal thickness maps with swept source optical coherence tomography. Systemic analysis included genotype, plasmatic biomarkers, [ferritin, chemokine ligand 18 (CCL18) and chitotriosidase (ChT)] and severity scoring systems results [“Gaucher Disease Severity Score Index Type I" (GauSSI-I) and “Gaucher disease severity scoring system” (GD-DS3)].

**Results:**

Nine subjects (18 eyes) were cases (female: 55.5%, mean age 45 years; male: 44.5%, mean age 36 years) and 40 subjects (80 eyes) were controls (female: 49%, mean age 50 years; male: 51%, mean age 55 years). There were no statistically significant differences when comparing ocular parameters (visual acuity; axial length, refractive errors, corneal parameters, lens, retinal and choroidal thickness) between case and control subjects (p>0.05). A statistically significant moderate correlation was observed between lower retinal thickness and choroidal quadrants thickness and greater disease severity scores. A lower central retinal thickness also correlates with higher biological plasmatic levels, and has a statistically significant association with the most affected patient with genotype N370S/Del 55pb. Conversely, higher pachymetry involves a more severe plasmatic concentration of biomarkers.

**Conclusions:**

Our results suggest that pachymetry, and retinal and choroidal thickness, are associated with burden biomarkers and disease severity index scores in Spanish patients with Gaucher’s disease Type I.

## Introduction

Gaucher’s disease (GD) is a rare autosomal recessive disease, within the group of lysosomal diseases, caused by mutations in the GBA gene that encodes the enzyme acid β-glucosidase, also known as β-glucocerebrosidase (EC 3.2.1.45). A total or partial deficiency of this protein results in severe lysosomal dysfunction in addition to the accumulation of glucosylceramide (GC) in macrophages of the reticuloendothelial system. GC represents the last link in the chain of degradation of complex lipids, in most part from the degradation of senescent haematological cell membranes. The disease mainly affects the liver, spleen and bone marrow. GD is a universal disease, with a worldwide prevalence of around 1/75,000 new-borns, but it is much more frequent in populations such as Ashkenazi Jews (prevalence between 1/400 − ½/500) [1]. In GD, the phenotypic expression and the clinical course of the disease are extremely heterogeneous, varying in severity among individuals as well as presenting with different degrees of involvement of different organs in the same individual. The most frequent clinical form is GD type I (OMIM 230800) (90% of patients) and is characterized by a marked variability in phenotypic expression with lack of neurological involvement (except for some cases with Parkinsonism) [2]. Nevertheless, there are some biomarkers (chemokine ligand 18 [CCL18] and chitotriosidase [ChT]) and severity score indexes which help clinicians manage this disease [3–5].

Gaucher cells can be also present in different ocular structures such as the conjunctiva, cornea, ciliary body, vitreous, inner surface of the retina, inner retinal layer, choroid and sclera [6]. As a result, studies have shown a high variety of structural and functional ocular abnormalities in GD which do not always affect visual acuity [6]; however, the relationship between ocular findings and the severity of the disease has not been investigated. The purpose of this study was to analyse ocular Spanish patients with GD Type I, and to investigate the possible correlation with phenotypic and disease-burden parameters.

## Material and methods

**Fig 1:**
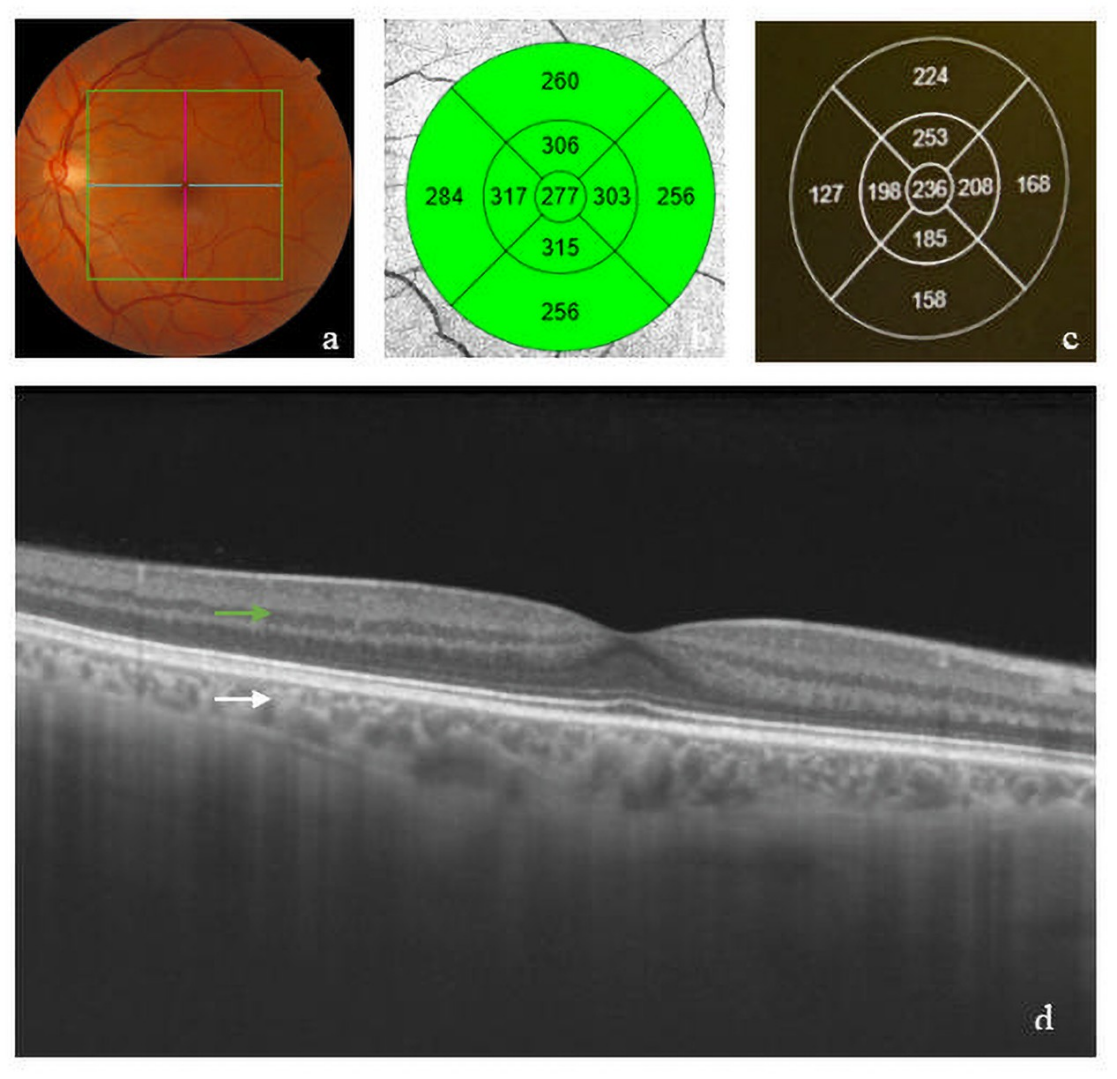
Macular SS-OCT in GD Type I. A.) macular grid in retinography; B.) retinal thickness map; C.) choroidal thickness map; D.) macular SS-OCT [white arrow] and retina [green arrow].

This cross-sectional observational study compared parameters belonging to 18 eyes from 9 Spanish patients with GD Type I with 80 eyes from 40 healthy controls. Both groups underwent a complete ophthalmological examination, including best-corrected visual acuity (BCVA) by using an Early Treatment Diabetic Retinopathy Study (ETDRS) chart, refractometry examination, ocular biometry, ocular motility test, slit-lamp biomicroscopy examination, and measurement of intraocular pressure by Goldmann applanation tonometry. We also performed a dilated fundus examination including indirect ophthalmic assessment with a 90-diopter lens or a fundus contact lens. Swept source optical coherence tomography (SS-OCT) was performed with the SS-OCT (DRI OCT Triton^™^ series, Topcon, Japan). Thickness maps were overlapped to the ETDRS grid (6 × 6 mm) to get values for each sector. Built-in software was used to automatically calculate thickness values in the ETDRS grid; the inner and outer rings, with semidiameters of 1,500 μm and 3,000 μm, respectively, were segmented into four quadrants (superior, inferior, nasal, and temporal). The central sector was defined as being within 1,000 μm from the foveal centre (Fig 1). From the medical history we obtained genotype, treatment and three plasmatic biomarkers: ferritin, CCL18 and ChT. We also included two clinical severity score index: “Gaucher Disease Severity Score Index Type I" (GauSSI-I) and “Gaucher disease severity scoring system” (GD-DS3). All subjects were recruited by the Unit of Rare Disorders and Ophthalmology department between May 2017 and May 2019 at the “Lozano Blesa” University Clinic Hospital in Zaragoza, Spain.

The research protocol adhered to Declaration of Helsinki, and the study obtained the approbation of the *local ethics committee* (*Comité Ético de Investigación Clínica de Aragón* [*CEICA*]). Informed consent was obtained from all studied subjects and, in the case of minors, their parents or the legally authorized representative.

Qualitative differences between the studied variables were compared by using Pearson’s chi-squared test. A normal distribution test was conducted by Kolmogorov-Smirnov test. As data showed a non-parametric distribution, a Mann-Whitney *U* test was employed to compare both groups. Pearson and Spearman Rank correlation coefficients were used for evaluating bivariate correlations. Statistical analysis data was completed using the SPSS software version 22.0 (IBM-SPSS, Inc, Chicago, IL, USA) and Stata 15.1. A p value of < 0.05 was considered statistically significant.

## Results

A total of 49 participants were included in this cross-sectional study. Nine subjects (18 eyes) were cases (female: 55.5%, mean age ± SD = 45 ± 12 years, [range 41–58]; male: 45.5%, mean age ± SD: 36 ± 19 years, [range 13–64]) and 40 subjects (80 eyes) were controls (female: 49%, mean age ± SD = 50 ± 13 years, [range 29–64]; male: 51%, mean age ± SD = 55 ± 15 years, [range 25–68]). No participant had evidence of any pathological ocular condition (neither cataracts, corneal alterations, retinopathy, glaucoma). Seven cases were on enzyme replacement therapy (ERT) and two on substrate reduction therapy (SRT), with a mean age at treatment initiation of 12.8 years (range 4–23 years). The most frequent genotype was N370S/N370S (55.5%; Table 1). Severity score index and activity of biomarkers are shown in Table 2. No significant differences were observed in ophthalmic parameters between cases and controls (Tables 3 and 4).

**Table 1:** Genotype frequency in patients with GD Type I.

Genotype	Patients (n = 9)	%
N370S/R120W	1	11.1
N370S/De155bpEx9	1	11.1
N370S/del2bp Ex10	1	11.1
N370S/N370S	5	55.5
N370S/R120W	1	11.1

**Table 2:** Biological parameters and severity score index in patients with GD Type I.

Variable	Mean	SD
**GAUSSI-I**	**3**	2.1
**GD DS3**	**1.1**	0.9
**ChT (mM/mL.h)**	**265.9**	363.7
**CCL18 (ng/mL)**	**81**	45.1
**FERRITIN (ng/mL)**	**513.6**	386.9

**Table 3:** Ocular variables: Anterior segment and biometric parameters.

Variable	Mean (± SD)		
	Patients	Controls	*p*
**Best-corrected visual acuity**	0.94 (±0.2)	1 (±0.0)	0.821
**Spherical power (diopters)**	-2.25 (±5.5)	-1.50 (±1.9)	0.712
**Cylinder power**	-1.05 (±0.9)	-2.32 (±1.9)	0.432
**Cylinder axis**	109 (±42.7)	97.2 (±60.1)	0.272
**Spherical equivalent**	-2.7 (±5.9)	-1.3 (±1.8)	0.354
**K1**	42.6 (±2.2)	43,0 (±0.9)	0.101
**K1 axis**	78 (±72.0)	82.2 (±77.1)	0.223
**K2**	43 (±2.7)	43.0 (±8.3)	0.167
**K2 axis**	81 (±44.0)	90.4 (±27.2)	0.881
**Endothelial cell density**	2589 (±257.1)	2718 (±347.7)	0.443
**Coefficient of variation**	29 (±3.41)	29 (±5.1)	0.291
**Cell hexagonality**	67.1 (±4.9)	67.9 (±5.5)	0.702
**Axial Length**	25.23 (±2.26)	23.43 (±0.9)	0.113
**Crystalline Lens Thickness**	4.11 (±0.36)	4.0 (±0.39)	0.642
**Anterior Chamber Depth**	3.34 (±0.46)	3.42 (±0.39)	0.251
**Pachymetry**	514 (±18.2)	518 (±12.1)	0.922

**Table 4:** Retinal and choroidal thickness (μm) by SS-OCT.

VARIABLE	Mean (± SD)		
	Patients	Controls	
**RETINA**			***p***
**Central**	246 (±35.0)	229 (±23.2)	0.293
**Inner temporal**	280 (±29.1)	232 (±32.4)	0.345
**Inner superior**	293 (±27.4)	312 (±31.3)	0.452
**Inner nasal**	293 (±41.2)	308 (±23.1)	0.742
**Inner inferior**	287 (±46.2)	310 (±20.7)	0.112
**Outer temporal**	242 (±21.3)	260 (±15.9)	0.321
**Outer superior**	260 (±15.6)	275 (±26.4)	0.431
**Outer nasal**	273 (±23.8)	294 (±33.1)	0.231
**Outer inferior**	242 (±33.2)	267 (1±8.9)	0.255
**CHOROID**			
**Central**	219 (±63.1)	305 (±68.2)	0.213
**Inner temporal**	216 (±73.2)	303 (±62.7)	0.432
**Inner superior**	250 (±75.4)	302 (±61.2)	0.234
**Inner nasal**	203 (±72.1)	282 (±66.2)	0.355
**Inner inferior**	204 (±55.8)	298 (±70.2)	0.321
**Outer temporal**	230 (±66.2)	292 (±57.4)	0.211
**Outer superior**	255 (±67.2)	296 (±62.1)	0.243
**Outer nasal**	193 (±44.6)	228 (±63.9)	0.122
**Outer inferior**	210 (±55.1)	284 (±66.3)	0.243

### Correlation between ocular parameters and severity score index

**Fig 2:**
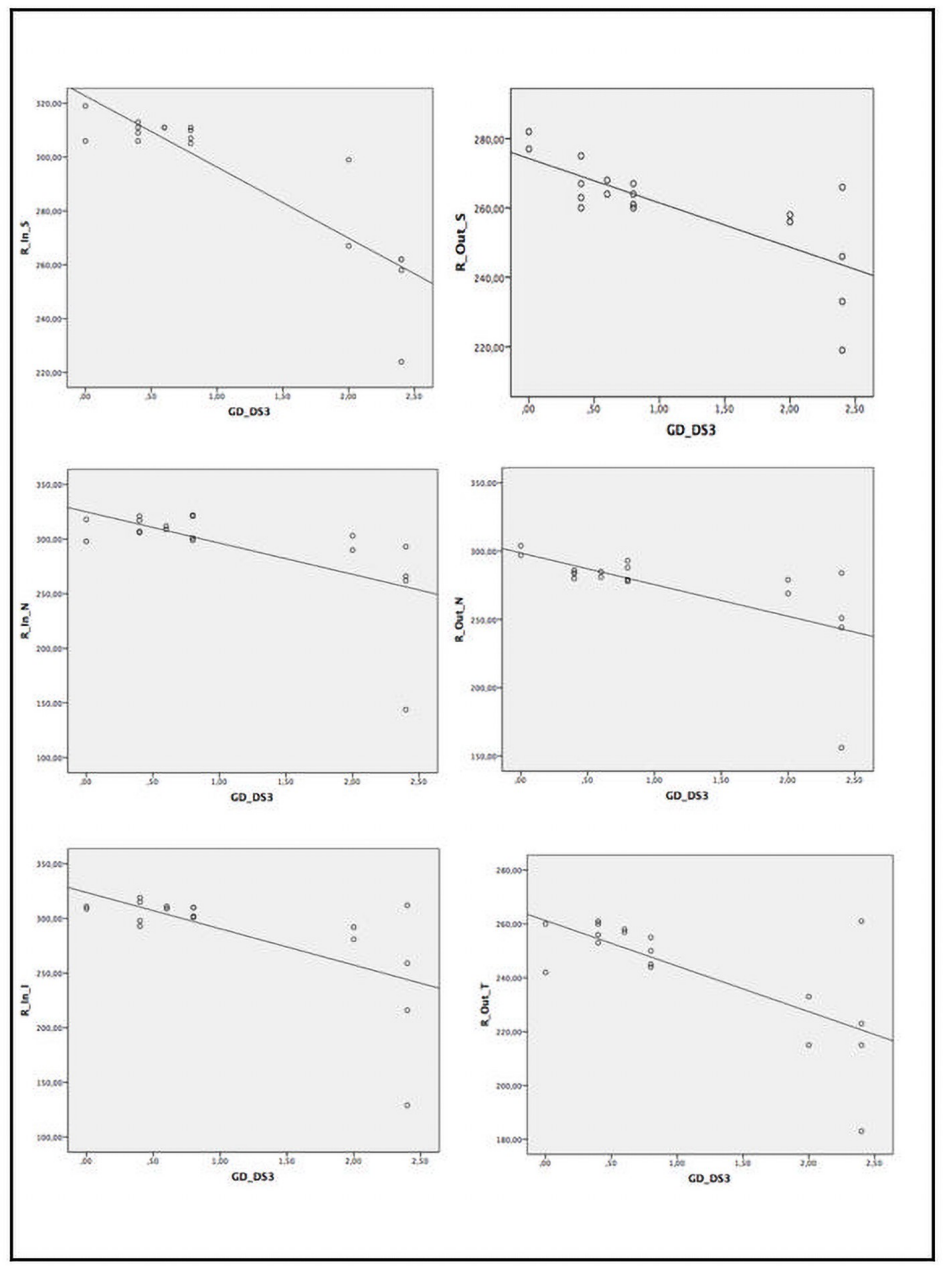
Moderate and strong correlation (r>0.6) between retinal thickness by SS-OCT and GD-DS3 score.

**Fig 3:**
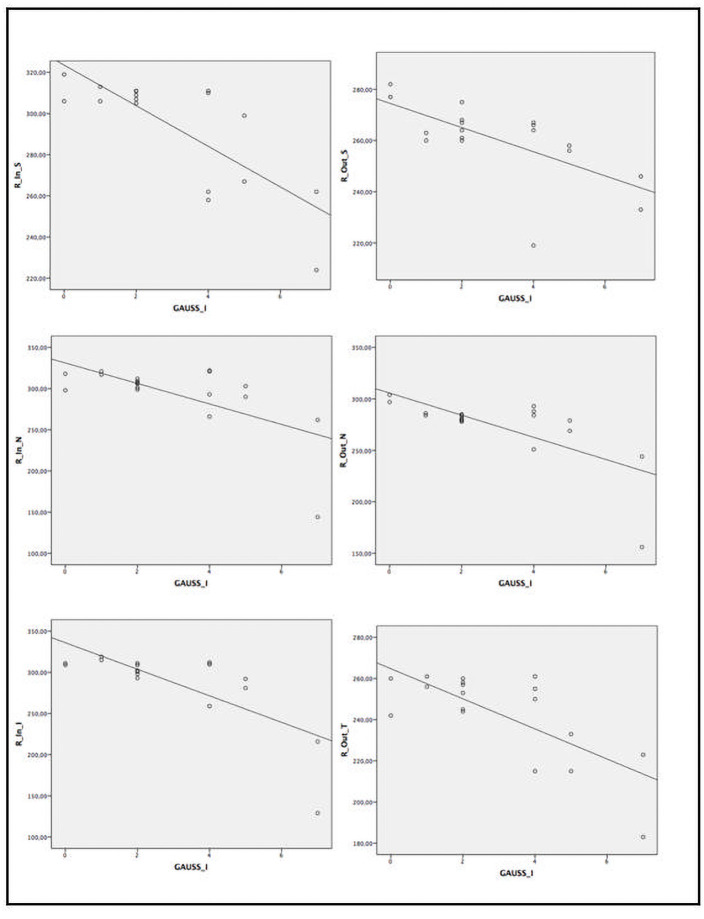
Moderate and strong correlation (r >0.6) between retinal thickness by SS-OCT and GauSSI-I score.

**Fig 4:**
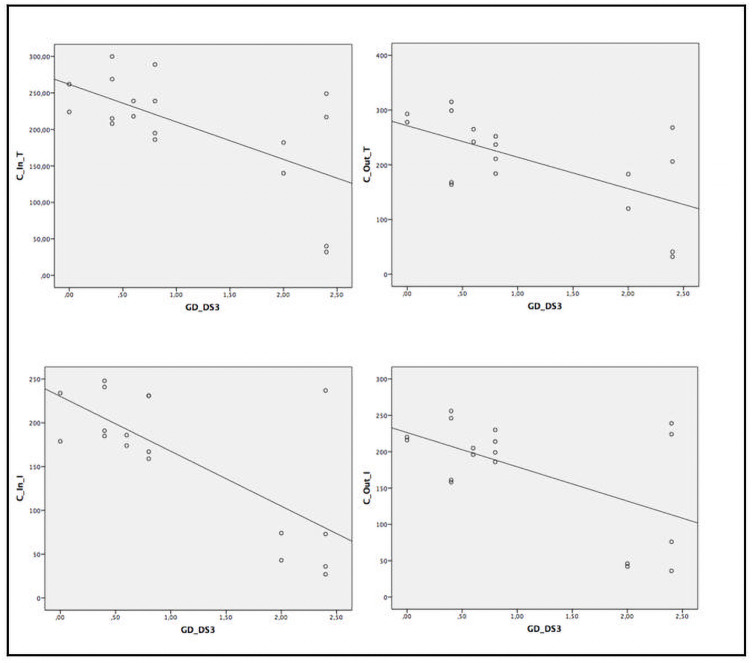
Moderate and strong correlation (r >0.6) between choroidal thickness by SS-OCT and GD-DS3 score.

**Fig 5:**
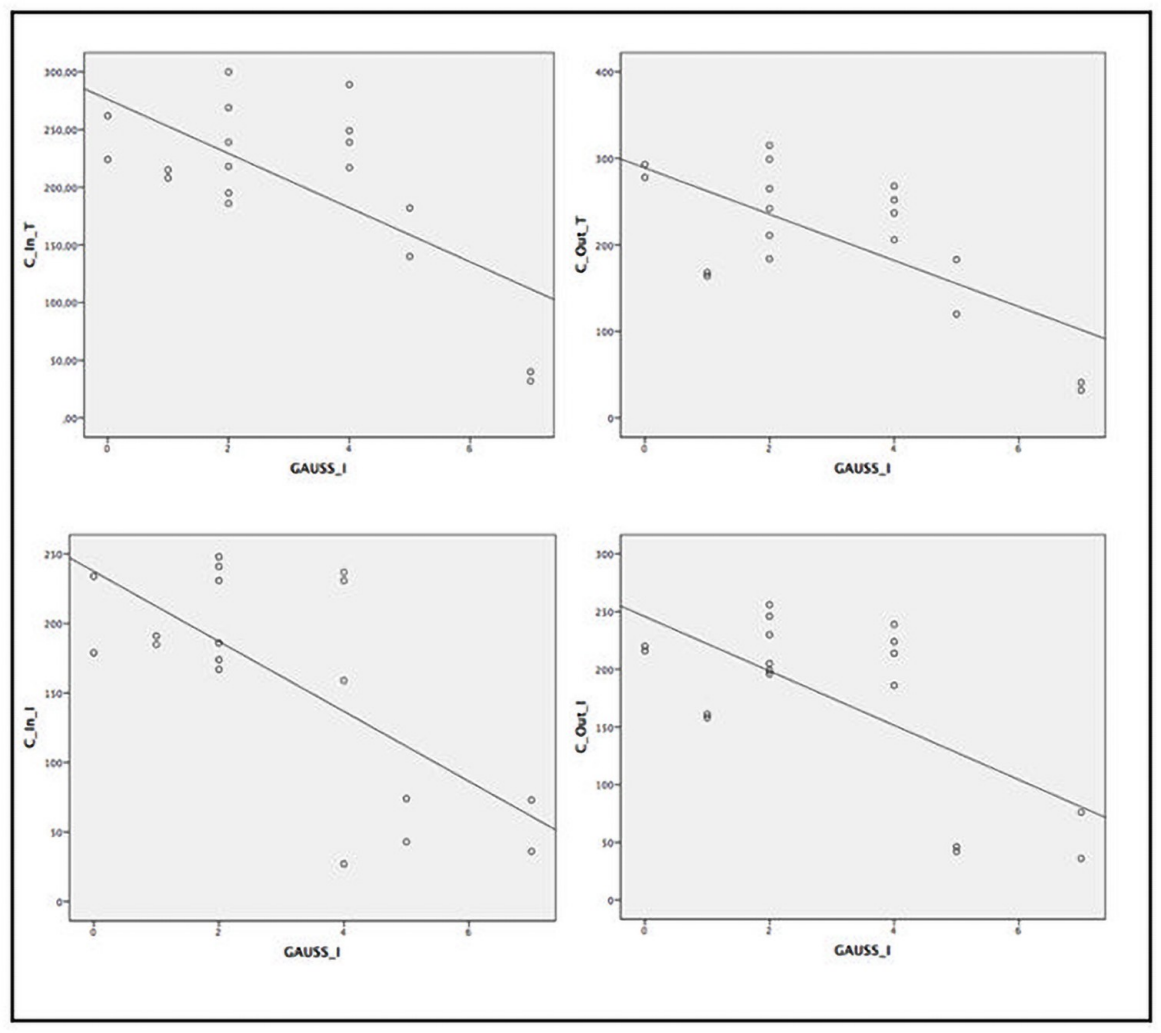
Moderate and strong correlation (r >0.6) between choroidal thickness by SS-OCT and GauSSI-I score.

When assessing the relationship between several ocular parameters and severity score indexes, we found statistically significant correlation between disease severity and SS-OCT parameters (Table 5 and Figs 2–5). A statistically significant moderate correlation was also observed (correlation coefficient [r] > 0.5; p<0.05) between lower retinal thickness and choroidal quadrant thickness and greater disease severity scores by GD-DS3 and GauSSI-I.

**Table 5:** Correlation coefficient (r) between severity score and retinal or choroidal thickness by SS-OCT. [Weak correlation (0.20–0.39); Moderate correlation (0.40–0.69); Strong correlation (0.70–0.99)], (p<0.05).

SS-OCT Thickness	GSD3 average	GAUSSI-I average
**Inner temporal**	**-0.610**	**-0,528**
**Inner superior**	**-0.777**	**-0.661**
**Inner nasal**	**-0.623**	**-0.640**
**Inner inferior**	**-0.637**	**-0.730**
**Outer temporal**	**-0.708**	**-0.723**
**Outer superior**	**-0.759**	**-0.661**
**Outer nasal**	**-0.717**	**-0.665**
**Outer inferior**	**-0.650**	**-0.592**
**Inner temporal**	**-0.629**	**-0.679**
**Inner superior**	**-0.354**	**-0.557**
**Inner nasal**	**-0.360**	**-0.466**
**Inner inferior**	**-0.730**	**-0,693**
**Outer temporal**	**-0.629**	**-0.690**
**Outer superior**	**-0.305**	**-0.352**
**Outer nasal**	**-0.135**	**-0.291**
**Outer inferior**	**-0.572**	**-0.676**

### Correlation between ocular parameters and biomarkers

When assessing the relationship between ocular parameters and activity of biomarkers, we found statistically significant correlations with corneal pachymetry measurements and central retinal thickness (Table 6). A higher pachymetry measurement involves a more severe plasmatic concentration of biomarkers. Conversely, a lower central retinal thickness correlates with higher biological plasmatic levels (r > 0.5; p <0.01). The ANOVA test showed a statistically significant (p < 0.05) association between retinal inner ring (temporal, nasal, and inferior) and genotype N370S/Del 55pb. A similar association was found between this genotype and retinal outer ring (nasal) (p < 0.05).

**Table 6:** Correlation coefficient (r) between ocular parameters and biomarkers’ activity. (p<0.05) [Weak correlation (0.20–0.39); Moderate correlation (0.40–0.69); Strong correlation (0.70–0.99)].

OCULAR PARAMETER	CCL18 (ng/mL)	ChT (mM/mL.h)
**Pachymetry**	**0.675**	**0.505**
**Central retinal thickness**	**-0.542**	**-0.542**

## Discussion

The clinical spectrum of GD ranges from lethal disease during infancy to symptom onset in late adulthood; the natural course of the disease is sometimes unpredictable and may progress at different rates in different organs [7].

Recent research reflects an increased effort to identify biomarkers that can be used for assisting clinicians in management of GD. Biomarkers in the eye have been studied in a range of different progressive diseases such as Alzheimer’s disease Parkinson’s disease (PD), using easily available, non-invasive ocular techniques [8, 9]. Literature indicates that retinal thinning (measured using OCT) may be used as an early biomarker for neurodegeneration and disease progression in PD [10, 11]. Patients with GD type I, are at a significantly increased risk for development of PD [12, 13]. Recently, ganglion cell layer (GCL) thinning measured by OCT has been demonstrated in the retinas of four patients with GD Type I who had clinical markers of potential early neurodegeneration in comparison vs controls [14]. This suggest that retinal thinning might be a biomarker of increased risk for developing a neurodegenerative disorder. Therefore, retinal assessment by OCT could represent a methodology for the early detection of PD in patients with GD that would allow them to benefit from early and timely treatment [15].

Retinal vascular ischaemia and retinal arterial sclerosis in GD type I patients have been reported in literature (results obtained at autopsy), and authors have hypothesized that retinal degeneration in these patients may have resulted from a reduction of glucocerebrosidase enzyme activity [16–18].

GD leads to impaired autophagy, storage of glucosylceramide, inflammatory processes, production of reactive oxygen species (ROS) and increased mitochondrial stress. Moreover, abnormalities in mitochondrial function and an increase of oxidative stress have been described with glucocerebrosidase inhibition [19, 20]. In this way, ERT has shown to be linked to improvements in autophagy-lysosomal pathway and a reduction in mitochondrial stress markers [21]. The role that mitochondrial oxidative stress plays in retinal degeneration diseases is well documented [22, 23]. The macula functions in high oxidative stress environment, which serve to further increase the stress placed on the macula itself [22]. Oxidative stress leads retinal pigment epithelium (RPE) dysfunction and chronic inflammation, which progress to retinal thinning and degeneration [24, 25]. The results of this current study provide evidence concerning the negative correlation between central retinal thickness and total disease burden expressed by higher concentrations of plasmatic biomarkers (CCL18 and ChT). ChT, a human chitinase synthesized by activated macrophages and CCL18 originating from disseminated Gaucher cells, has shown a strong correlation with GD activity. Further to this, literature support a clear correlation between disease activity and several other clinical parameters of GD showing their usefulness as circulating biomarkers for disease progression [26–28].

The fact that, in our data series, we report a case of GD Type I with associated PD in a patient with genotype (N370S / Delta55), which is recognised as being the genotype a.) most commonly associated with more severe disease, and b.) associated with the greatest degree of ocular involvement, support the usefulness of practicing ocular studies for the early screening of this neurodegenerative entity.

Conversely, a direct correlation between thicker cornea and higher concentrations of plasmatic biomarkers (CCL18 and ChT) has been also found. The value of the cornea as a potential marker for lipid-related disorders has been previously reported [29].

Researchers have also described corneal abnormalities in patients with GD Type I. A study of GD carriers with keratoconus revealed the presence of keratocytes with changes to the rough endoplasmic reticulum and alterations in the lipid profile vs healthy controls. [30]. Ueno et al. demonstrated an increased central corneal thickness in a child with GD Type I [31]. Geens et al. described corneal features of a 57-years-old man with GD Type I as assessed by in vivo confocal microscopy; they demonstrated focal zones of thickening in corneal stroma, destroyed stromal architecture, folds in the Descement membrane and a thickened central corneal as a result [32].

Authors who examined ophthalmic histopathological and immunohistochemical features and ultrastructural changes in two patients with GD Type I (results obtained at autopsy) reported calcium deposits on the Bowman’s membrane and a duplicated epithelial basement membrane with a thickened cornea as a result [17]. Although corneal opacities associated with glucosylceramide accumulation in keratinocytes have been widely observed in GD Type III, our findings and literature review also suggest corneal implications in GD Type I.

The need for a methodology that is specific and sensitive to assess and monitor disease severity and progression led to the creation of the disease severity scores systems. GD-DS3 is a composite measure of disease burden and correlates well with a clinical global impression scale for monitoring the response to treatment [5]. GAU-SSI-1, which is more complex than GD-DS3 and requires specialized technology, is considered the most valuable tool for evaluating clinical and phenotypical severity in GD [4, 33]. A negative correlation between them and retinal or choroidal thickness by SS-OCT was found. More accurate measurement of choroidal thickness have been made possible through a higher wavelength light SS-OCT vs conventional OCT, meaning SS-OCT provides better visualization, offers deeper penetration into RPE, improves our knowledge of choroidal structures, and performs automated segmentation of the choroidal boundaries [34].

In current literature, only a limited number of study cases have shown alterations in the vascular layer: two have shown choroidal sclerosis, one neovascularization [17, 35]. We are the first study measuring the choroid in patients with GD Type I. Since a wide variety of haematological alterations have been found in patients with GD Type I [36, 37], we speculate that a possible vascular component also exists in the eye.

Ocular imaging techniques used to measure corneal, retinal or choroidal thickness (such as pachymetry or SS-OCT) represent a non-invasive, inexpensive, and rapid methodology that can be used to improve clinical examination in GD. In our study, we did not find significant differences between cases and controls, but studies with a larger sample size might find some differences. Another limitation of this study is its cross-sectional design as the primary objective was explorative in regard to the association between ocular parameters and phenotypical features of GD Type I.

## Conclusions

In conclusion, this is the first study to report an association between ocular parameters with burden biomarkers and disease severity index scores in patients with GD type I. Longitudinal studies should be developed to confirm the correlation between ocular parameters and disease severity to further evaluate the value of these parameters in guiding treatment decisions in patient with GD Type I.

## Supporting information

S1 File Dataset. (XLSX)
